# Computed tomography aspects of thoracic metastases from osteosarcoma: pictorial essay

**DOI:** 10.1590/0100-3984.2022.0107-en

**Published:** 2023

**Authors:** Jéssica Albuquerque M. Silva, Bruno Hochhegger, Viviane Brandão Amorim, Gláucia Zanetti, Edson Marchiori

**Affiliations:** 1 Instituto Nacional de Câncer (INCA), Rio de Janeiro, RJ, Brazil; 2 University of Florida, Gainesville, FL, USA; 3 Universidade Federal do Rio de Janeiro (UFRJ), Rio de Janeiro, RJ, Brazil

**Keywords:** Neoplasm metastasis, Lung neoplasms/secondary, Osteosarcoma/secondary, Tomography, X-ray computed, Metástase neoplásica, Neoplasias pulmonares/secundário, Osteossarcoma/secundário, Tomografia computadorizada

## Abstract

Osteosarcoma is the most common primary bone tumor, with a higher incidence in the second decade of life, and it often leads to pulmonary metastases. The most common pattern seen on computed tomography is one of multiple well-defined nodules in the lung parenchyma, often with calcifications. Because of the variety of presentations of pulmonary metastases in osteosarcoma, including atypical forms, knowledge of the computed tomography aspects of these lesions is important for characterizing and evaluating the extent of the disease, as well as for distinguishing metastatic disease from other benign or malignant lung diseases. This essay discusses the main tomographic findings of pulmonary metastases from osteosarcoma.

## Abstract (pt)

O osteossarcoma é o tumor ósseo primário mais comum, com maior incidência na segunda década de vida, sendo as metástases pulmonares achado frequente. O padrão tomográfico mais comum das metástases pulmonares de osteossarcoma é o de múltiplos nódulos bem definidos no parênquima pulmonar, frequentemente com calcificações. Em razão da multiplicidade de apresentações das metástases pulmonares do osteossarcoma, inclusive com formas atípicas, o conhecimento dos aspectos dessas lesões na tomografia computadorizada do tórax é importante para a caracterização e avaliação da extensão da doença, além de permitir a diferenciação entre doença metastática e outras doenças pulmonares benignas ou malignas. Este ensaio discute os principais achados tomográficos das metástases pulmonares de osteossarcoma.

## INTRODUCTION

Osteosarcoma is the most prevalent primary bone tumor, accounting for approximately 20% of all such tumors. Its highest incidence occurs among individuals between 20 and 30 years. Initial metastases from an osteosarcoma are characteristically hematogenous. At the time of diagnosis, microscopic metastases are present in almost all patients, clinically detectable in 15-20%. The lung is the organ most commonly affected, with metastases detected in approximately 80% of cases^(1-3)^.

Despite the development of new combined treatments for osteosarcoma (neoadjuvant therapy and surgery), relapse occurs in approximately 75% of patients with lung metastases at diagnosis and in 30-40% of those initially diagnosed with nonmetastatic disease. Recurrence is associated with a poor prognosis, especially where there are lung lesions. The overall survival rate among patients with nonmetastatic (localized) osteosarcoma is 60-70%, compared with 10-30% among those with metastatic disease^(1-3)^.

Chest computed tomography (CT) is the preferred imaging method for assessing lung metastases, enabling characterization of disease extent and associated complications.

While most lung metastases from osteosarcoma appear as nodular and calcified, up to 40% are non-calcified and atypical findings are not uncommon. Identifying these atypical radiological presentations could be the key to increasing the accuracy of CT in this context.

On CT, most pulmonary metastases from osteosarcoma present as multiple, well-defined, rounded nodules of varying sizes, predominantly in the lower portions of the lung parenchyma. However, such lesions can exhibit a variety of atypical presentations, including cavitation, distribution in uncommon lung locations, a micronodular pattern, hemorrhagic metastases, tumor thrombi, calcified or noncalcified mediastinal and hilar lymph node enlargement, lymphangitic carcinomatosis, and pneumothorax^(1-7)^.

Radiologists’ knowledge of atypical presentations is essential for differentiating between metastatic disease and other malignant or benign conditions.

## TOMOGRAPHIC MANIFESTATIONS

**Figure 1:**
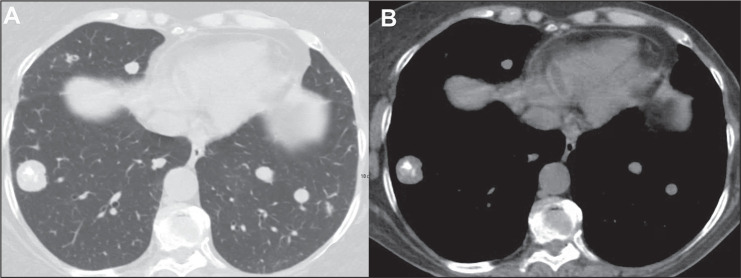
Nodular metastases in a 72-year-old woman with a primary osteosarcoma of the femur. Axial CT slices with a lung window (A) and a mediastinal window (B), showing multiple nodules of varying sizes in both lungs, the largest nodule being in the right lung and containing foci of calcification.

**Figure 2:**
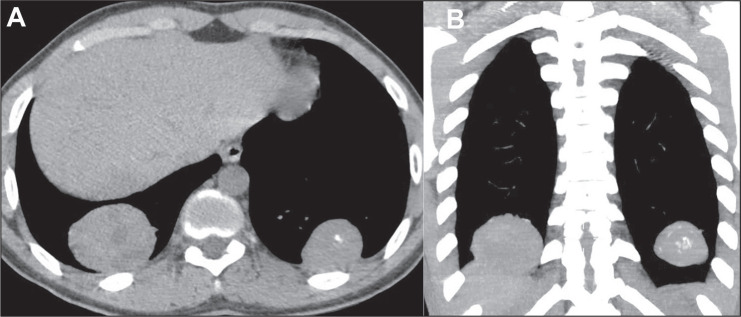
Masses in a 16-year-old male patient with a primary osteosarcoma of the tibia. Axial and coronal CT slices (A and B, respectively) with a mediastinal window, showing bilateral masses in the lower lobes, with foci of calcification on the left.

**Figure 3:**
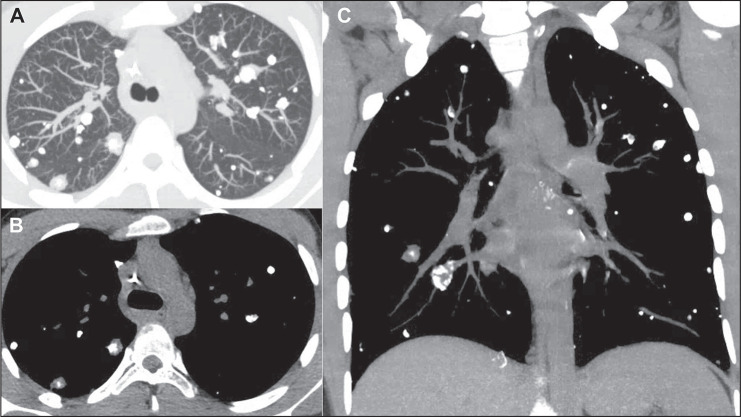
Calcified nodular metastases in a 15-year-old male patient with a primary osteosarcoma of the femur. Axial CT slices with a lung window (A) and a mediastinal window (B), together with a coronal CT slice with a mediastinal window (C), showing multiple calcified nodules in both lungs. The metastases were diagnosed by biopsy, and the patient evolved to death in eight months.

**Figure 4:**
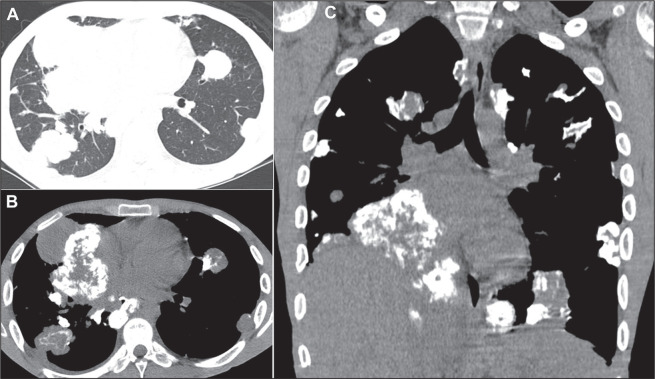
Calcified metastases in a 14-year-old male patient with a primary osteosarcoma of the femur. Axial CT slices with a lung window (A) and a mediastinal window (B), together with a coronal CT slice with a mediastinal window (C), showing calcified masses and nodules in both lungs, with a reduction in the volume of the right lower lobe.

The most common aspect seen on CT scans of patients with pulmonary metastases from osteosarcoma is the presence of multiple nodules (Figure 1), which may also be accompanied by masses (Figure 2). Calcifications within pulmonary nodules generally suggest a benign nature, often corresponding to granulomas or, less commonly, hamartomas. However, calcification or ossification can also occur within metastatic nodules. Certain tumors, including sarcomas and carcinomas, especially osteosarcomas, synovial sarcomas, chondrosarcomas, and mucinous or papillary adenocarcinomas, have the potential to produce calcified metastases. In some instances, metastases from osteosarcomas manifest as multiple calcified nodules or masses (Figures 3 and 4), mimicking granulomatous disease^(1-7)^.

**Figure 5:**
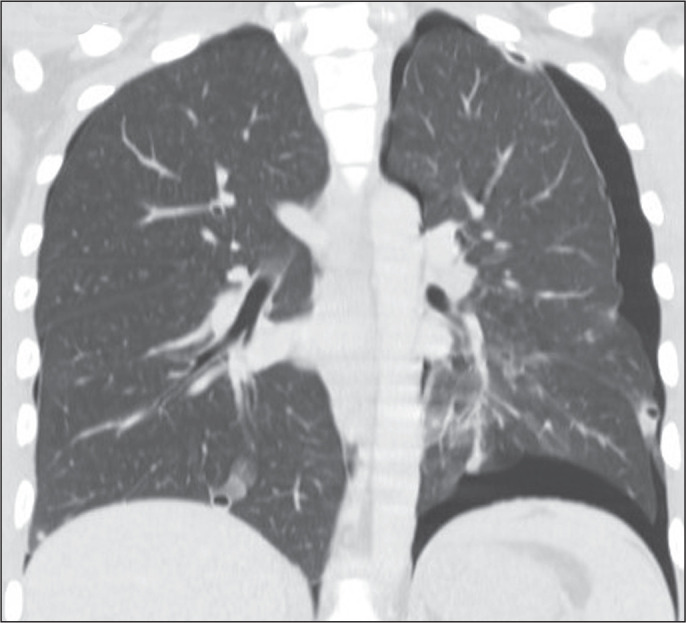
Cavitary metastases with pneumothorax in a 16-year-old male patient with a primary osteosarcoma of the femur. Coronal CT scan with a lung window, acquired at the time of diagnosis, showing multiple cavitary lung lesions together with bilateral pneumothorax.

Pulmonary metastases from osteosarcoma can also be cavitary (Figure 5), presumably as a result of tumor necrosis induced by chemotherapy or by the behavior of the neoplastic lesion itself. Cavitation can also occur through a check-valve mechanism caused by tumor infiltration of bronchial structures. Pneumothorax is a common complication in such cases, caused by the formation of a bronchopleural fistula resulting from tumor necrosis. Therefore, in patients diagnosed with osteosarcoma and presenting with spontaneous pneumothorax, occult pulmonary metastases should be investigated^(1-3,7)^.

**Figure 6:**
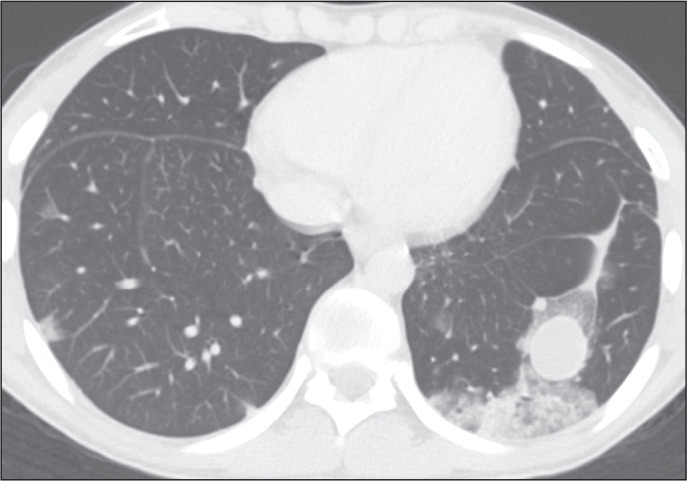
Hemorrhagic metastasis in a 16-year-old male patient with a primary osteosarcoma of the femur. Axial CT slice with a lung window, showing nodular opacity, surrounded by ground-glass opacities, in the left lower lobe, suggestive of hemorrhage.

Hemorrhagic lung metastases are found in lesions that present fragility of the neovascular tissue, leading to vessel rupture. Such metastases typically present as nodular opacities with a ground-glass halo (the halo sign) or with diffusely ill-defined margins (Figure 6). Although the halo sign is nonspecific, its presence in patients diagnosed with an associated malignant neoplasm should raise suspicion^(7)^.

**Figure 7:**
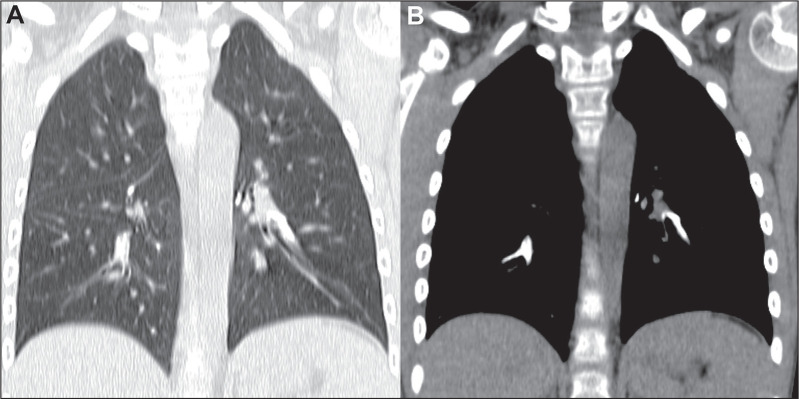
Vascular metastases in a 7-year-old boy with a primary osteosarcoma of the femur. Coronal CT slices with a lung window (A) and a mediastinal window (B), showing bilateral calcified intravascular pulmonary metastases in the descending branches of the pulmonary arteries, confirmed by biopsy.

**Figure 8:**
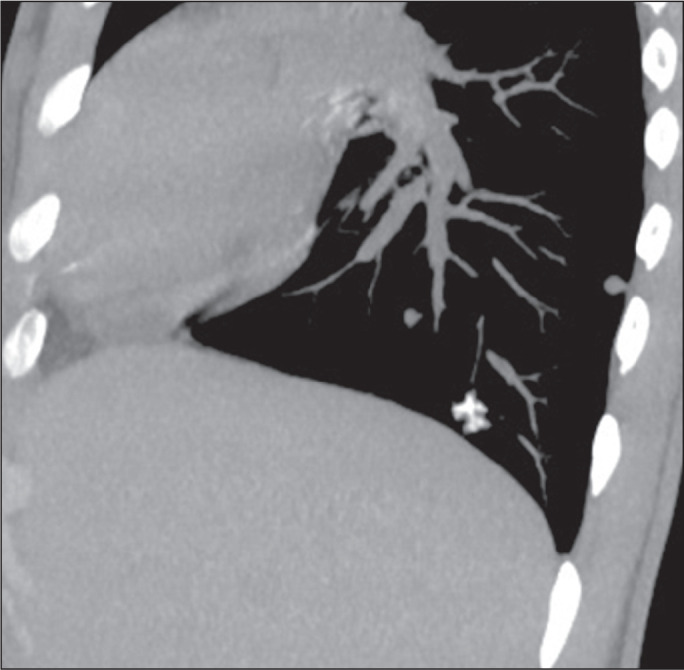
Intravascular metastases in a 15-year-old male patient with a primary osteosarcoma of the tibia. Sagittal CT reconstruction showing calcified opacities with a tree-in-bud pattern in the right lower lobe.

Intravascular pulmonary tumor emboli can be seen in patients who are asymptomatic or have nonspecific respiratory symptoms, potentially leading to delayed diagnosis or even going undiagnosed. Additionally, intravascular pulmonary metastases are typically located in small or medium-sized arteries, which makes their radiological diagnosis challenging. Typical findings on pulmonary angiography include filling defects in segmental arteries, with subsegmental arteries less commonly affected, and occasionally accompanied by calcifications (Figure 7). CT scan typically shows enlarged arteries with lobulated contours, as well as peripheral areas of pulmonary infarcts. A tree-in-bud appearance can also be present and, if calcified (Figure 8), strongly suggests metastases from osteosarcoma^(4)^.

Endobronchial metastases from osteosarcoma are rare and tipically occur simultaneously with lesions in the lung parenchyma. The most common radiological aspect is atelectasis of a lung lobe or of an entire lung, along with an endobronchial nodule^(1-3)^.

**Figure 9:**
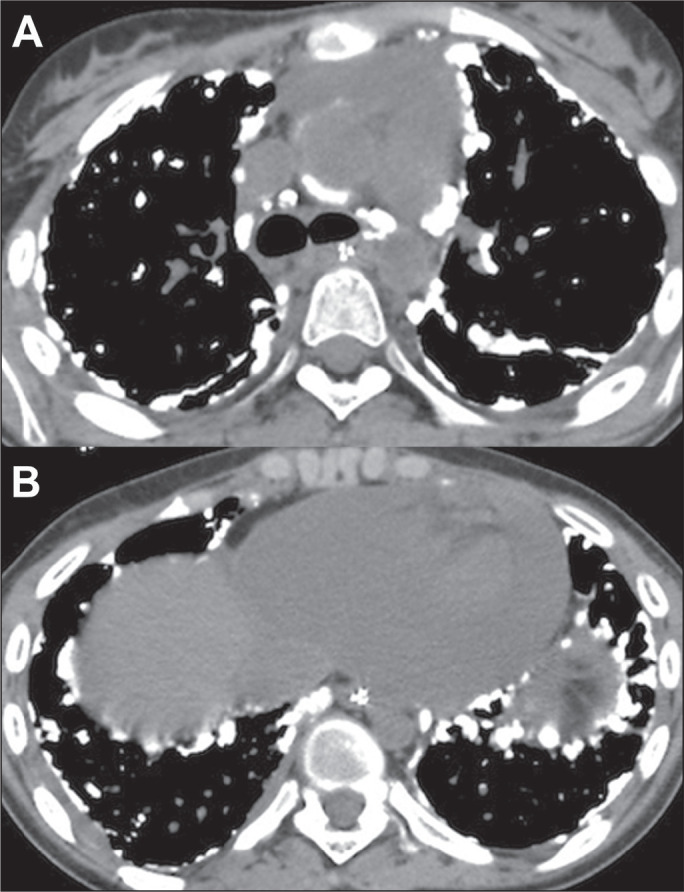
Tumor dissemination via the lymphatic system in a 14-year-old female patient with a primary osteosarcoma of the femur. CT, with a mediastinal window, of the upper and lower lung fields (A and B, respectively), showing several calcified nodules in the subpleural regions and along the fissures. Note also the mediastinal lymph node calcifications and pericardial effusion.

**Figure 10:**
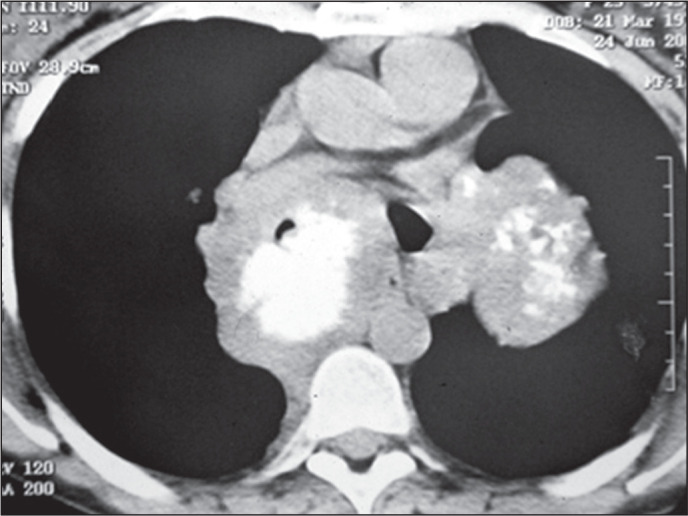
Lymph node metastases in a 13-year-old female patient with a primary osteosarcoma of the tibia. Axial CT scan with a mediastinal window, showing a mass with foci of calcification in the left lung, along with a large mass with coarse calcification in the subcarinal region, corresponding to enlarged calcified lymph nodes.

The combination of lymphangitic carcinomatosis and osteosarcoma is uncommon. Retrograde tumor invasion and extension into the lymphatic and perilymphatic interstitium can lead to dissemination of tumor cell throughout interlobular septa, fissures, and pleural surfaces, without nodular lung metastasis. These lesions, distributed throughout the pulmonary lymphatic system, can exhibit calcifications (Figure 9). As illustrated in Figure 10, metastatic lymph node involvement can also be observed, presenting as lymph node calcifications^(5)^.

**Figure 11:**
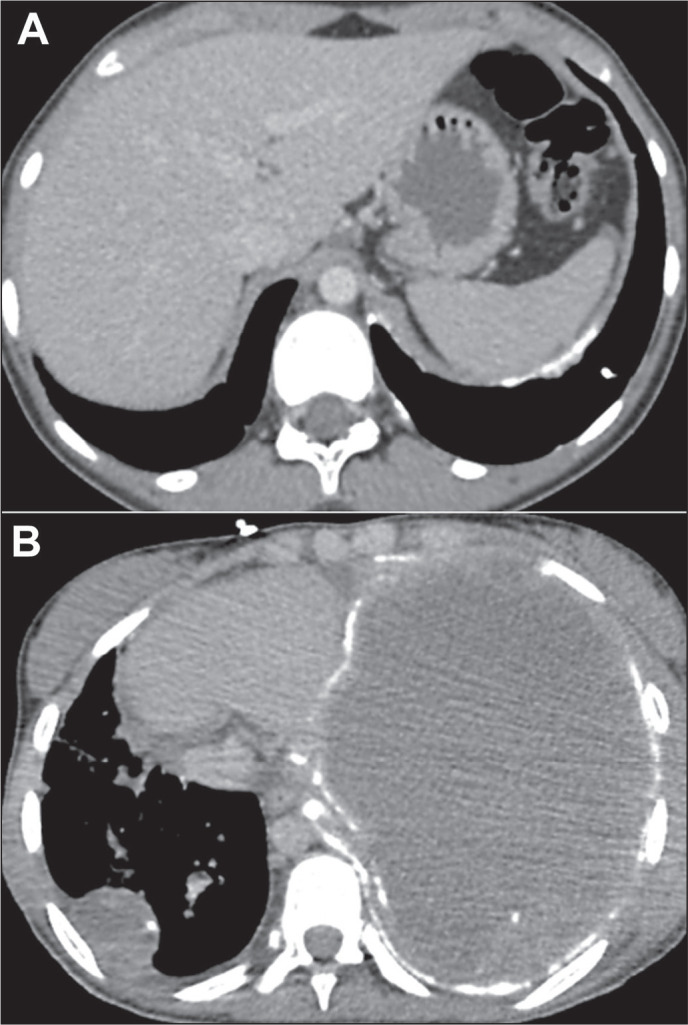
Pleural metastases in a 16-year-old female patient with a primary osteosarcoma of the tibia. Axial CT slice (A) showing small nodules and calcified pleural plaques on the left. Axial CT slice acquired one month later (B) showing substantial worsening of the disease, with complete opacification of the left hemithorax caused by a large heterogeneous mass, together with pleural thickening and calcification, which occupies the entire pleural cavity, as well as a mass with calcification on the right.

While the lung is the most common site of hematogenous metastasis from osteosarcoma, pleural metastases can occur in rare cases. Isolated pleural metastasis, in the absence of lung implants, is also uncommon. The CT appearance shows diffuse pleural thickening, often with calcifications^(6)^, as demonstrated in Figure 11.

## CONCLUSION

There is a wide spectrum of manifestations of thoracic metastases from osteosarcoma. On imaging exams, metastases from osteosarcoma can appear indistinguishable from other infectious, inflammatory, or neoplastic thoracic diseases. Although the typical forms are highly suggestive of the diagnosis, radiologists should be familiar with the wide variety of atypical presentations of these lesions.
